# An Expanded Oct4 Interaction Network: Implications for Stem Cell Biology, Development, and Disease

**DOI:** 10.1016/j.stem.2010.03.004

**Published:** 2010-04-02

**Authors:** Mercedes Pardo, Benjamin Lang, Lu Yu, Haydn Prosser, Allan Bradley, M. Madan Babu, Jyoti Choudhary

**Affiliations:** 1 Proteomic Mass Spectrometry, Wellcome Trust Sanger Institute, Hinxton, Cambridgeshire CB10 1SA, UK; 2 Mouse Genomics, Wellcome Trust Sanger Institute, Hinxton, Cambridgeshire CB10 1SA, UK; 3 MRC Laboratory of Molecular Biology, Cambridge CB2 0QH, UK

**Keywords:** STEMCELL

## Abstract

The transcription factor Oct4 is key in embryonic stem cell identity and reprogramming. Insight into its partners should illuminate how the pluripotent state is established and regulated. Here, we identify a considerably expanded set of Oct4-binding proteins in mouse embryonic stem cells. We find that Oct4 associates with a varied set of proteins including regulators of gene expression and modulators of Oct4 function. Half of its partners are transcriptionally regulated by Oct4 itself or other stem cell transcription factors, whereas one-third display a significant change in expression upon cell differentiation. The majority of Oct4-associated proteins studied to date show an early lethal phenotype when mutated. A fraction of the human orthologs is associated with inherited developmental disorders or causative of cancer. The Oct4 interactome provides a resource for dissecting mechanisms of Oct4 function, enlightening the basis of pluripotency and development, and identifying potential additional reprogramming factors.

## Highlights

► Oct4 associates with a large varied set of proteins ► Half of Oct4's partners are regulated by key stem cell transcription factors ► Most Oct4 binding proteins are required for early development in mouse ► Some Oct4 partners are associated with human developmental disorders or cancer

## Introduction

Two characteristics define embryonic stem cells (ESCs), self-renewal ability and pluripotency. Recently, ectopic expression of combinations of transcription factors (Oct4, Nanog, Sox2, c-Myc, Esrrb, and Klf4) has been shown to reprogram mouse and human fibroblasts into a pluripotent state (Kaji et al., 2009, Okita et al., 2007, Takahashi et al., 2007, Takahashi and Yamanaka, 2006, Woltjen et al., 2009, Yu et al., 2007). The induced pluripotent stem cells (iPSCs) are very similar to ESCs and retain the ability to self-renew and differentiate into the three germ layers and thus promise great therapeutic potential in regenerative medicine (Amabile and Meissner, 2009, Maherali et al., 2007, Wernig et al., 2007). Despite the recent flurry of studies, our understanding of the molecular mechanisms and players that drive ESC self-renewal and differentiation is still limited.

The POU transcription factor Oct4, also termed Pou5f1, is a central player in ESC self-renewal and differentiation into specific lineages. Levels of Oct4 must be tightly regulated to maintain the ESC status. A decrease in Oct4 levels by 50% induces differentiation toward the trophectoderm lineage, whereas a 50% increase causes differentiation into mesoderm and endoderm (Niwa et al., 2000, Shimozaki et al., 2003). Oct4 plays an essential role in early development given that loss of Oct4 in the mouse embryo causes the failure of the inner cell mass to develop (Nichols et al., 1998).

Oct4 regulates transcriptional programs to maintain ESC pluripotency primarily in collaboration with transcription factors Sox2 and Nanog (Boyer et al., 2005, Chew et al., 2005, Pan et al., 2006). Several genome-wide analyses of regulatory targets of key pluripotency factors has led to the identification of sets of jointly regulated or bound targets, highlighting a complex transcriptional circuitry responsible for ESC maintenance (Babaie et al., 2007, Boyer et al., 2005, Ivanova et al., 2006, Kim et al., 2008, Loh et al., 2006, Matoba et al., 2006). Also recently, various other factors have been functionally linked to Oct4 and Nanog, after identification of their binding partners by affinity purification and mass spectrometry (Wang et al., 2006, Liang et al., 2008). These studies have revealed a compact regulatory module responsible for ESC pluripotency (Orkin et al., 2008).

To further elucidate the ESC transcriptional network, we have carried out an unbiased and extensive study of Oct4-associated proteins, using an affinity purification and mass spectrometry approach. In contrast with a previous similar study (Wang et al., 2006), epitope-tagged Oct4 was expressed under the control of Oct4's endogenous promoter to keep the natural transcriptional regulation. The epitope tagging strategy circumvents the need for specific antibodies and facilitates a generic purification procedure that results in cleaner and higher yield samples than traditional immunoprecipitation experiments. Our data significantly expands the current repertoire of Oct4-associated proteins, thereby shedding light on the complex regulatory circuitries of ESCs. The Oct4 interactome provides a useful resource to investigate the mechanisms of Oct4 function and regulation and to explore the basic principles underlying stem cell biology.

## Results

### Efficient FTAP Tagging of *Oct4* by Recombineering and Single-Copy BAC Transgenesis

To investigate the molecular network around Oct4/Pou5f1, we used an epitope-tagging affinity purification strategy. We modified the SPA tag (Zeghouf et al., 2004) containing the 3× FLAG epitope and a calmodulin binding peptide (CBP) separated by a TEV cleavage site, by adding an extra TEV site to improve cleavage efficiency (Figure S1A available online). The FTAP was fused at the C terminus of the *Oct4* coding region by recombineering into a BAC clone containing full-length *Oct4*. This was then integrated into the *Hprt* locus of ESCs by recombinase-mediated cassette exchange (RMCE) (Prosser et al., 2008). The whole procedure is depicted in Figure S1B. Expression levels of the Oct4-FTAP fusion protein were ∼30% that of endogenous Oct4 expressed from two alleles (Figure S1C), close to what should be expected given that it is expressed from an extra copy of the gene and avoiding interference with the ESC phenotype, as shown by the expression of ESC markers by the transgenic clone (Figure S1C).

### Identification of Oct4-Associated Proteins

**Figure 1:**
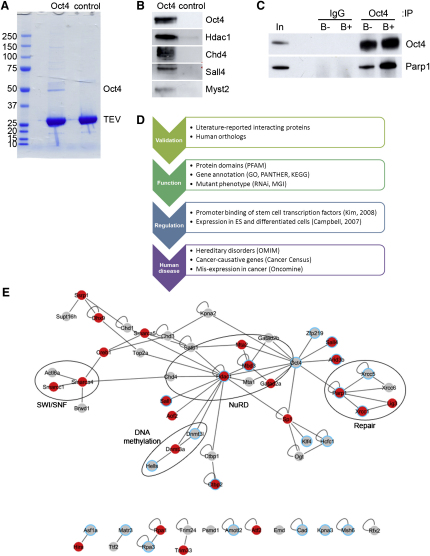
Analysis of Oct4-Interacting Proteins (A) Typical Oct4-FTAP and control purifications. Molecular weight markers (kDa) are shown. (B) Western blots confirming some of the interacting proteins identified by mass spectrometry. C, Western blot showing co-immunoprecipitation of endogenous Parp1 with Oct4 in the presence (B+) or absence (B-) of benzonase. In denotes whole cell extract. D, Workflow tracing the systems analyses of the Oct4 interactome. E, Network of protein-protein interactions within the Oct4 dataset. Blue circles are proteins downregulated upon ES cell differentiation. Red fill indicates proteins whose absence results in embryonic lethality in the mouse. See also Figure S1.

The tandem affinity tag allows single- and double-affinity purifications. We first performed three independent one-step purifications on whole-cell extracts from both Oct4-FTAP-expressing and control unmodified cells (Figure 1A). Eluates were separated by gel electrophoresis, and whole lanes were excised into several regions, digested, and analyzed by nano-liquid chromatrography/tandem mass spectrometry (LC-MS/MS). MS results files from each lane were merged and searched against IPI with Mascot. The data is available in the PRIDE database (Martens et al., 2005) (www.ebi.ac.uk/pride). The data was converted with the PRIDE Converter (Barsnes et al., 2009) (http://code.google.com/p/pride-converter). The criteria for peptide and protein identification are detailed in Experimental Procedures. Mass spectrometry analysis resulted in the identification of 92 proteins (excluding Oct4 itself) that were present in all Oct4-FTAP purifications, but not in controls (Table 1). The identification of some of the interacting proteins was confirmed by Western blotting (Figure 1B). These data considerably expand the list of published Oct4 binding partners and represent a major extension of the sets reported in two similar studies (Liang et al., 2008, Wang et al., 2006). We detected 13 previously identified Oct4 interacting proteins in our study (Table S6). These included Sall4, Arid3b, Zfp219, and Sp1 (Wang et al., 2006), Kpna2 (Li et al., 2008), Parp1 (Gao et al., 2009), and NuRD complex members Hdac1, Mta1/2, and Gatad2a/b (Liang et al., 2008, Wang et al., 2006). Furthermore, we also identified Sox2 and Nanog, two of the best characterized Oct4 binding partners (Ambrosetti et al., 1997, Chew et al., 2005, Liang et al., 2008, Wang et al., 2006), and Zfp281, Requiem/Dpf2, Yy1, RYBP, Dax1, Esrrb, and Arid3a, recently shown to physically interact with Oct4 (Donohoe et al., 2009, Sun et al., 2009, van den Berg et al., 2008, Wang et al., 2006, Wang et al., 2008) in one or two (Arid3a and Esrrb) purifications, but because of our strict criteria of result reproducibility, we did not include them in the final data set. We also identified proteins reported to be linked to Oct4 through association with some of its interactors, namely Sall1 and Smarcc1 (Wang et al., 2006). Eight previously identified Oct4-interacting proteins were either not detected, namely EWS, NF45, Cdk1 (Wang et al., 2006), and Zfp206 (Yu et al., 2009), or found also in controls, such as beta-catenin (Takao et al., 2007), Hdac2 (Liang et al., 2008), Ctcf (Donohoe et al., 2009), and Wwp2 (Xu et al., 2009, Xu et al., 2004).

**Table 1:** Oct4-Associated Proteins Classified into Protein Complexes and/or Functional Categories

Complex/Protein Class	Gene Name	Accession	Description	MW	Exp I	Exp II	Exp III
Bait	Pou5f1	IPI00117218	POU domain, class 5, transcription factor 1	38705.35	20	19	21

**NuRD Complex**							

	Chd4	IPI00396802	chromodomain-helicase-DNA-binding protein 4	219096.34	21	40	33
	Gatad2a	IPI00625995	p66 alpha isoform a	67762.39	13	11	16
	Gatad2b	IPI00128615	isoform 1 of transcriptional repressor p66-beta	65712.08	13	7	16
	Mbd3	IPI00131067	isoform 1 of methyl-CpG-binding domain protein 3	32168	4	6	4
	Mta1	IPI00624969	Mta1 protein	80019.75	13	14	16
	Mta2	IPI00128230	metastasis-associated protein MTA2	75723.93	25	17	21
	Mta3	IPI00125745	isoform 1 of metastasis-associated protein MTA3	67719.08	10	9	7
	Hdac1	IPI00114232	histone deacetylase 1	55609.93	11	12	11

**Spalt-like Transcriptional Repressors**							

	Sall1	IPI00342267	Sal-like 1	141745.1	11	17	18
	Sall3	IPI00123404	isoform 1 of Sal-like protein 3	140610.62	5	5	6
	Sall4	IPI00475164	isoform 1 of Sal-like protein 4	114711.29	35	29	28

**BAF Complex**							

	Smarca4	IPI00875789	SWI/SNF related, matrix associated, actin dependent regulator of chromatin, subfamily a, member 4, isoform CRA_b	181913.68	3	5	1
	Smarcc1	IPI00125662	isoform 1 of SWI/SNF complex subunit SMARCC1	123326.28	7	3	4
	Actl6a	IPI00323660	actin-like protein 6A	47930.54	4	1	1

**FACT Complex**							

	Ssrp1	IPI00407571	isoform 2 of FACT complex subunit SSRP1	81766.73	36	12	32
	Supt16h	IPI00120344	FACT complex subunit SPT16	120319.5	64	34	56

**LSD1 Complex**							

	Aof2	IPI00648295	amine oxidase (Flavin containing) domain 2	95113.5	9	6	7
	Rcor2	IPI00226581	REST corepressor 2	58042.89	5	6	2

ISWI Chromatin Remodeling Complex	Smarca5	IPI00396739	SWI/SNF-related matrix-associated actin-dependent regulator of chromatin subfamily A member 5	122291.43	5	7	2

**INO80 Chromatin-Remodeling Complex**							

	Ino80	IPI00378561	isoform 1 of putative DNA helicase INO80 complex homolog 1	177265.25	5	6	1
	Nfrkb	IPI00274469	nuclear factor related to kappa-B-binding protein	139134.5	22	11	4
	Actl6a	IPI00323660	actin-like protein 6A	47930.54	4	1	1

**Histone Chaperone Complex**							

	Asf1a	IPI00132452	histone chaperone ASF1A	23099.19	3	1	3
	Cabin1	IPI00380107	calcineurin binding protein 1	245584.2	8	18	7
	Hira	IPI00123694	isoform long of protein HIRA	113235.4	10	7	10
	Ubn2	IPI00854896	isoform 4 of uncharacterized protein KIAA2030	142523.51	17	19	11

**Transcription Factors**							

	Arid3b	IPI00277032	isoform 1 of AT-rich interactive domain-containing protein 3B	61091.99	9	8	3
	Atf2	IPI00110172	isoform 1 of cyclic AMP-dependent transcription factor ATF-2	52550.73	4	5	5
	Creb1	IPI00119924	isoform 1 of cAMP response element-binding protein	36879.65	3	3	3
	Ctbp1	IPI00128155	isoform 1 of C-terminal-binding protein 1	48170.7	9	4	3
	Ctbp2	IPI00856974	isoform 2 of C-terminal-binding protein 2	107801.19	10	3	4
	Klf4	IPI00120384	Kruppel-like factor 4	52531.81	1	1	2
	Mitf	IPI00125758	isoform A of microphthalmia-associated transcription factor	59160.39	2	3	1
	Nfyc	IPI00108204	nuclear transcription factor Y subunit gamma	37230.86	7	3	1
	Sp1	IPI00323887	isoform 1 of transcription factor Sp1	81309.84	5	4	6
	Tcfe3	IPI00380308	isoform 1 of transcription factor E3	61555.7	9	5	7
	Tcfeb	IPI00314502	transcription factor EB	52638.18	2	3	2
	Zbtb10	IPI00223276	zinc finger and BTB domain containing 10 isoform 1	118071.48	9	5	12
	Zbtb2	IPI00652356	putative uncharacterized protein	58189.61	1	1	1
	Zbtb43	IPI00230530	zinc finger protein 297B isoform a	57551.37	2	1	3
	Zfhx3	IPI00475055	AT motif binding factor 1	410697.11	3	36	1
	Zfp217	IPI00758403	zinc finger protein 217	115181.65	11	10	4
	Zfp219	IPI00469594	zinc finger protein 219, isoform CRA_a	78831.33	4	6	7
	Zfp513	IPI00830836	isoform 1 of Zinc finger protein 513	59968.12	1	2	1
	Zic2	IPI00127145	zinc finger protein ZIC 2	55546.36	1	1	1
	Zscan4b	IPI00755380	similar to Gene model 397	58667.99	4	6	7

**Regulation of Transcription**							

	Acin1	IPI00121136	isoform 1 of apoptotic chromatin condensation inducer in the nucleus	151000.03	12	2	13
	Brwd1	IPI00121655	isoform A of bromodomain and WD repeat-containing protein 1	262057.12	1	2	2
	Hcfc1	IPI00828490	host cell factor C1	216798.82	19	8	12
	Ifi202b	IPI00126725	interferon-activable protein 202	50727.44	4	6	3
	Phf17	IPI00453799	isoform 1 of Protein Jade-1	95434.25	4	2	4
	Rfx2	IPI00406298	DNA-binding protein RFX2	76998.5	8	1	1

General Transcription	Ttf2	IPI00112371	transcription termination factor 2	126706.43	5	2	2

**Recombination/Repair**							

	Lig3	IPI00124272	isoform Alpha of DNA ligase 3	114656.59	5	6	1
	Msh6	IPI00310173	MutS homolog 6	152813.37	10	4	5
	Parp1	IPI00139168	putative uncharacterized protein	113491.6	24	17	33
	Top2a	IPI00122223	DNA topoisomerase 2-alpha	173567.4	30	44	12
	Xrcc1	IPI00118139	DNA repair protein XRCC1	69270.68	6	3	1
	Xrcc5	IPI00321154	ATP-dependent DNA helicase 2 subunit 2	83802.29	6	5	13
	Xrcc6	IPI00132424	ATP-dependent DNA helicase 2 subunit 1	69726.04	12	5	12

**Replication**							

	Rpa1	IPI00124520	replication protein A 70 kDa DNA-binding subunit	69620.87	2	3	2
	Rpa3	IPI00132128	replication protein A 14 kDa subunit	13688.99	1	1	1

**Helicases**							

	Chd1	IPI00107999	chromodomain-helicase-DNA-binding protein 1	197601.13	8	9	7
	Chd3	IPI00675483	chromodomain helicase DNA binding protein 3	234196.54	5	6	6
	Chd5	IPI00875673	chromodomain helicase DNA binding protein 5 isoform1	224027.21	10	9	11
	Dhx9	IPI00339468	isoform 2 of ATP-dependent RNA helicase A	150907.1	15	15	31
	Hells	IPI00121431	isoform 1 of lymphocyte-specific helicase	95806.47	3	1	1

**Histones**							

	Hist1h3e	IPI00282848	histone cluster 2, H3c1 isoform 2	20348.12	8	9	4
	Hist1h4b	IPI00407339	histone H4	11360.38	11	13	6
	Hist3h2bb	IPI00229539	histone cluster 3, H2bb	17248.15	9	7	4

**Heterogeneous Nuclear Ribonucleoproteins**							

	Hnrnpab	IPI00277066	heterogeneous nuclear ribonucleoprotein A/B isoform 1	36302.44	3	2	2
	Hnrnpl	IPI00620362	heterogeneous nuclear ribonucleoprotein L	64550.49	10	4	8
	Hnrnpu	IPI00458583	putative uncharacterized protein	88661.02	8	2	12

**Histone Ubiquitination (E3 Ubiquitin Ligase Complex)**							

	Cul4b	IPI00224689	Cullin 4B	111314	7	3	9
	Ddb1	IPI00316740	DNA damage-binding protein 1	128026.73	7	5	16

**Enzymes**							

	Cad	IPI00380280	carbamoyl-phosphate synthetase 2, aspartate transcarbamylase, and dihydroorotase	245649.59	4	18	11
	Dnmt3a	IPI00131694	isoform 1 of DNA (cytosine-5)-methyltransferase 3A	103203.53	4	4	7
	Dnmt3l	IPI00109459	DNA (cytosine-5)-methyltransferase 3-like	49159.08	7	3	7
	Myst2	IPI00228457	isoform 2 of histone acetyltransferase MYST2	67588.54	4	2	3
	Ogt	IPI00420870	isoform 1 of UDP-N-acetylglucosamine–peptide N-acetylglucosaminyltransferase 110 kDa subunit	118131.41	14	4	10
	P4ha1	IPI00399959	isoform 2 of prolyl 4-hydroxylase subunit alpha-1	61132.82	14	5	5
	Ppp2r1a	IPI00310091	Serine/threonine-protein phosphatase 2A 65 kDa regulatory subunit A alpha isoform	66079.23	3	4	1
	Trim24	IPI00227778	isoform short of transcription intermediary factor 1-alpha	114824.79	3	4	6
	Trim33	IPI00409904	isoform alpha of E3 ubiquitin-protein ligase TRIM33	125931.28	2	2	2

**Karyopherins**							

	Kpna2	IPI00124973	Importin subunit alpha-2	58234.28	7	7	2
	Kpna3	IPI00230429	Importin subunit alpha-3	58193	2	1	2

Chaperones	Dnaja1	IPI00132208	DnaJ homolog subfamily A member 1	45580.73	6	1	5

Proteasome	Psmb6	IPI00119239	proteasome subunit beta type-6	25590.57	2	2	1

**Nuclear Assembly/Organization**							

	Emd	IPI00114401	Emerin	29417.38	2	1	1
	Matr3	IPI00453826	Matrin-3	95085.04	14	6	11

**Miscellaneous**							

	Amotl2	IPI00263333	isoform 1 of Angiomotin-like protein 2	85454.32	1	4	2
	Cubn	IPI00889898	Cubilin	407679.63	3	7	2
	Nudc	IPI00132942	nuclear migration protein nudC	38334.29	2	2	2

The number of unique peptides for three independent experiments is shown. MW, molecular weight. See also Table S6.

We next performed tandem affinity purification, although yields were not high because of the low levels of tagged Oct4 and purification efficiency. We identified seven proteins of the 92, mainly members of NuRD, Sall proteins, and transcription factors E3 and EB (Table S6). We believe these constitute the highest-affinity interactors, given that they can endure a more stringent purification. Although yielding small numbers of interactors, this purification is still at the level of previous similar studies (Liang et al., 2008, Wang et al., 2006).

For confirmation, we immunoprecipitated endogenous Oct4 from whole-cell extracts of untagged feeder-free E14 mouse ESCs in duplicate and analyzed immunoprecipitates by mass spectrometry. Forty-six proteins reproducibly overlapped with the FTAP data set (Table S6). We detected all proteins identified in a similar experiment (Liang et al., 2008). Not surprisingly, proteins that were reproducibly copurified with endogenous Oct4 tended to be more abundant in our single-affinity purification data set.

To address whether the interactions detected were due to coassembly of factors on chromatin, we then immunoprecipitated Oct4 in the presence of DNase treatment with benzonase. Western blotting showed that Parp1, a ubiquitous DNA-binding protein, coimmunoprecipitates with Oct4 even in the absence of DNA (Figure 1C). Preliminary purification experiments with a differently tagged Oct4-FTAP cell line suggested that other DNA-binding proteins such as ligase 3 and topoisomerase 2a also copurify with Oct4 in the absence of DNA (M.P. and S.P. Shen, data not shown). This suggests that the interactions we detect are not DNA mediated.

Summing up, over 50% of the Oct4-associated proteins (47 of 92) varying in abundance across our data set have been confirmed by independent means, suggesting that the data set we provide here is a bona fide set of Oct4 binding partners.

### Functional Annotation Analysis of Oct4-Associated Proteins

To uncover general trends in the functions of the Oct4-interacting proteins, we carried out computational systems-level analyses in a workflow depicted in Figure 1D. We first performed a functional annotation analysis using DAVID 2008 (Dennis et al., 2003) and the PANTHER database (Thomas et al., 2003). We found an enrichment of GO terms such as nucleus, chromosome, and chromatin in the cellular component ontology; nucleic acid binding, protein binding, transcription factor activity, in the molecular function ontology; and transcription, regulation of gene expression, and embryonic development in the biological process ontology (Figure S2 and Table S2). This indicates that GO terms associated to Oct4 are highly represented within the list of Oct4-copurifying proteins, adding consistency to the data set. Twenty proteins in the data set (21%) are annotated with the GO term “transcription factor activity.” Oct4 has been shown to associate with several transcription factors, and our results agree with the notion that combinatorial binding among pluripotency factors, which contributes to achieving specificity in gene regulation, may be a frequent pattern in ESCs (Chambers and Tomlinson, 2009).

We also analyzed the enrichment of proteins involved in cellular pathways. DAVID analysis detected an enrichment of proteins involved in the control of gene expression by vitamin D nuclear hormone receptor, mainly members of the FACT and SWI/SNF complexes. The data set also contains several proteins involved in the nuclear part of the Wnt signaling pathway, as revealed by PANTHER analysis. The Wnt pathway is involved in stem cell maintenance (Anton et al., 2007, Sato et al., 2004), possibly by modulating levels of pluripotency factors Oct4, Nanog, and Sox2 (Kalmar et al., 2009).

We then analyzed the domain composition of Oct4-interacting proteins and detected a significant abundance of DNA-binding and chromatin-related domains (Tables S3 and S4). Highly represented domains were DEAD/DEAH box helicase, SNF2-related, PHD and zinc fingers, and chromo, bromo, and homeobox domains, all of which are either involved in ATP-dependent chromatin remodeling or bind DNA and posttranslationally modified nucleosomes, thereby influencing gene expression.

The data set was manually classified into known protein complexes and functional categories. As shown in Table 1 and supported by the GO and PANTHER analyses, Oct4 associates mainly with transcriptional regulators, but also with a variety of other chromatin binding proteins involved in DNA replication, recombination, and repair, proteins involved in nuclear assembly and/or organization, and diverse enzymes, some of which are responsible for addition of posttranslational modifications.

To gain an overall view of the previously known interactions among Oct4-associated proteins, we retrieved interaction data from INTACT, HRPD, and MINT for the data set and represented them as a protein interaction network (Figure 1E). The network comprises 80 known interactions for 57 of the proteins including Oct4. Repressor complexes NuRD and SWI/SNF and DNA repair and de novo DNA methylation modules are apparent in the network.

### Transcriptional Regulation of Oct4-Associated Proteins

**Figure 2:**
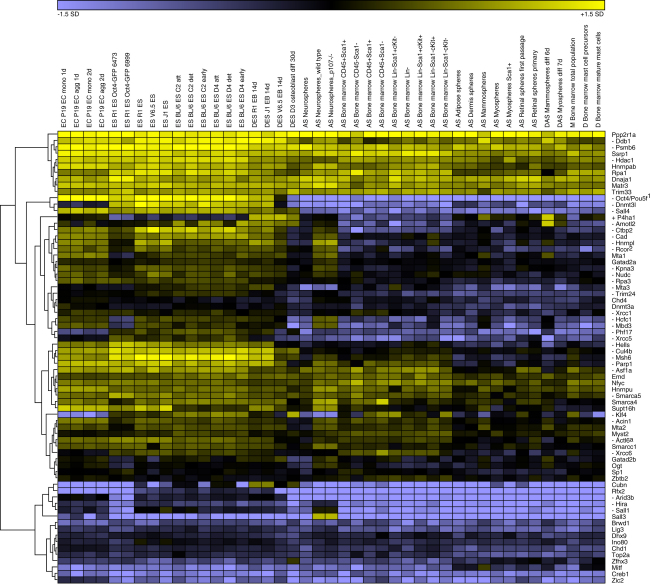
Expression of Oct4-associated proteins in ESCs and Differentiated Cell Types Based on Microarray Data Columns correspond to experimental samples, arranged as follows: embryonal carcinoma P19 (EC), ES cells (ES), differentiating embryonic stem cells (DES), adult stem cells (AS), differentiated adult stem cells (DAS), mixed cells (M), and differentiated cells (D). Average-linkage hierarchical clustering was performed to arrive at the final layout. Genes whose expression is significantly up or downregulated in differentiation are marked.

Many known Oct4 binding proteins are ESC-specific factors (Wang et al., 2008). However, Oct4 has also been shown to interact with more general modulators of transcription that are expressed ubiquitously, such as members of the NuRD complex (Liang et al., 2008, Orkin et al., 2008, Wang et al., 2006). We investigated the patterns of expression of the Oct4-associated data set in cells at different stages of differentiation, including embryonic carcinoma, embryonic stem cells, embryoid bodies, and various differentiated cell types, on the basis of transcriptomics data (Campbell et al., 2007). Protein abundances were fairly varied and most interactors maintained near constant expression across the samples analyzed (Figure 2). This suggests that Oct4 interacts mostly with proteins that are ubiquitously expressed in both differentiated and undifferentiated cells. After statistical analysis, 33 Oct4-interacting proteins were found to be significantly less expressed in differentiated cells compared to ESCs, in correlation with Oct4's expression pattern (Figure 2 and Table S6). Among these are the DNA methylation regulatory factor Dnmt3l and the developmentally important transcription factors Klf4, Sall1, and Sall4. We observed that many complexes or interacting pairs in the interaction network contained at least one member significantly downregulated upon ESC differentiation (Figure 1E), possibly conferring an ESC-specific role.

**Figure 3:**
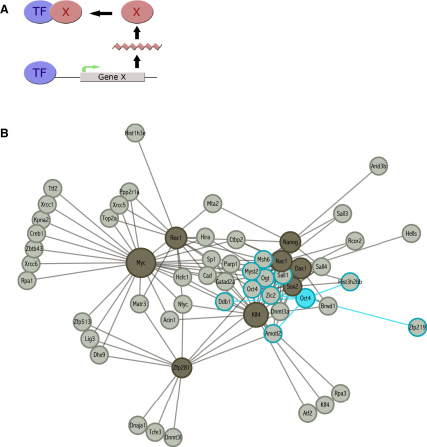
Transcriptional Regulation of Oct4-Associated Proteins (A) Scheme of transcription factor feedback regulation. (B) Regulatory network of targets of ESC transcription factors among Oct4 partners. Stem cell transcription factors and their target genes among Oct4-associated proteins are shown in dark and light gray, respectively. Oct4 and its regulatory targets are highlighted in blue. See also Figure S3.

Regulation of gene expression involves complex dynamics employing sequence-specific DNA binding proteins that form the transcriptional regulatory network (Babu et al., 2004, Jothi et al., 2009, Luscombe et al., 2004). Transcription factors often operate in feedback loops, whereby the expression of a transcriptional target modulates the function of the transcription factor itself (Figure 3A). Pluripotency factors in ESCs are no exception and show a high degree of transcriptional auto and interregulation (Orkin et al., 2008). We next investigated whether the promoters of the Oct4-associated gene set contain binding sites for transcription factors that are central in the establishment and maintenance of ESC identity. Promoter binding sites for nine such transcription factors have previously been identified by ChIP-on-chip (Kim et al., 2008). These include Oct4, Dax1, Klf4, c-Myc, Nac1, Nanog, Rex1, Zfp281, and Sox2.

Nine of the 92 Oct4-associated proteins were found to be transcriptionally regulated by Oct4 itself in mouse ESCs, and 51% of genes encoding Oct4 partners are targets of at least one key ESC transcription factor (Figure S3). This concurs with findings by others on a much smaller data set (Orkin et al., 2008, Wang et al., 2006). To assess whether this is statistically significant, we compared the results to 1000 randomly generated sets of 92 proteins. The expected percentage of promoter binding by ESC transcription factors was only 28% (Z = 4.45, p < 10^−15^), indicating that it is a significant trait of the data set. Several genes in the interaction set are common targets of multiple transcription factors (20 of 92 are targets of at least three transcription factors), making it likely that they have central roles in pluripotency and self-renewal. Ten of these are significantly downregulated in differentiation, and all but two show a downregulation trend (Table S6), in agreement with the hypothesis that genes bound by multiple factors are active in ESCs and become repressed as cells differentiate (Chambers and Tomlinson, 2009, Kim et al., 2008, Orkin et al., 2008). We constructed a regulatory network by integrating promoter target data for the nine stem cell transcription factors with the list of Oct4 binding proteins (Figure 3B). Several of the transcription factors cluster together because of shared targets (e.g., Sox2, Nanog, Nac1, and Dax1), whereas c-Myc and Klf4 exclusively target certain groups of Oct4-interacting factors. This agrees with the genome-wide trend of the c-Myc target set, which is largely distinct from the rest of the pluripotency factors (Kim et al., 2008).

### Role of Oct4 Interactome in Mouse Embryonic Stem Cell Biology and Development

We next explored the consequences of loss of Oct4-interacting proteins in ESCs or mouse development. Five Oct4-interacting proteins have been identified as required for stem cell self-renewal in large scale RNAi screens (Ding et al., 2009, Hu et al., 2009). Literature searches ascribed a role in ESC self-renewal or pluripotency to an additional nine Oct4-interacting proteins (Table S6).

**Figure 4:**
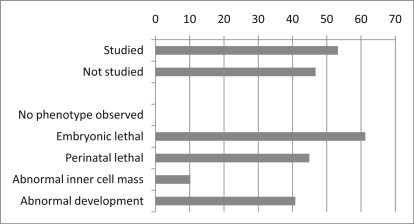
Distribution of Phenotypes Caused by Mutations in the Genes Encoding Oct4-Associated Proteins Numbers are percentage of genes studied. Full data are shown in Table S5.

Loss-of-function phenotypes in mice were available in the MGI database for 49 Oct4-associated proteins. All 49 show diverse phenotypes when absent or mutated (Figure 4 and Table S5). Significantly, 83% (41 of 49) of the studied knockout alleles of the interaction set showed embryonic and/or perinatal lethality, with over 60% (30 of 49) being embryonic lethal (Figure 1E). Similar analyses on random control data sets allowed us to conclude that the result is significant (Z = 7.48, p < 10^−15^). In addition, 41% (20 of 49) showed an abnormal development phenotype. These results indicate a high level of requirement for components of the Oct4 network in early mouse development. Although feedback loops are expected to add to the robustness of a transcriptional regulatory network, the high frequency with which mutation of single Oct4 partners causes severe early developmental phenotypes suggests they are essential downstream regulatory hubs.

### Involvement of Oct4-Interacting Proteins in Human Disease and Cancer

Given the extent of their part in mouse development and the current excitement about the cancer stem cell hypothesis, we next explored a possible role of Oct4-associated proteins in human disease. Human orthologs were identified for all Oct4-associated proteins and sequence identities determined between mouse and human (Figure S4). All Oct4-associated proteins were found to be highly conserved, with a median sequence identity of 94%, compared to 77% genomic median. This strong sequence conservation implies that the findings reported here could be applied to human ESC biology.

We next investigated the involvement of the human orthologs in human disease and development of cancer interrogating the OMIM database and the Cancer Gene Census, which records genes whose mutation has been causally linked to cancer. Genes encoding 14 of 92 Oct4-associated proteins are implicated in one or more hereditary diseases, mostly of developmental nature, with six of them predisposing to certain types of cancer (Table 2). Somatic mutations in eight Oct4-associated proteins and Oct4 itself were found to be responsible for different types of cancer, often through gene translocations, presumably affecting their regulation (Table 3). Statistical analysis on random sets indicated that the observed numbers of Oct4-interacting proteins linked to human disease (Z = 1.06, p < 10^−15^) and cancer (Z = 4.43, p < 10^−15^) are significantly higher than expected.

**Table 2:** Genetic Disease Associations of Oct4-Interacting Proteins

Gene	Disorder Type	Disorder
CREB1	cancer	histiocytoma, angiomatoid fibrous, somatic
CUBN	hematological	megaloblastic anemia-1, Finnish type
CUL4B	multiple	mental retardation syndrome, X-linked, Cabezas type mental retardation-hypotonic facies syndrome, X-linked, 2
EMD	muscular	Emery-Dreifuss muscular dystrophy
MATR3	muscular	myopathy, distal 2
MITF	multiple	Tietz syndrome Waardenburg syndrome, type IIA Waardenburg syndrome/ocular albinism, digenic
MSH6	cancer	colorectal cancer, hereditary nonpolyposis, type 5 endometrial cancer, familial mismatch repair cancer syndrome
SALL1	multiple	Townes-Brocks branchiootorenal-like syndrome Townes-Brocks syndrome
SALL4	multiple	Duane-radial ray syndrome IVIC syndrome
TFE3	cancer	renal cell carcinoma, papillary, 1
TRIM24	cancer	thyroid carcinoma, papillary
TRIM33	cancer	thyroid carcinoma, papillary
ZFHX3	cancer	prostate cancer, susceptibility to
ZIC2	developmental	holoprosencephaly-5

See also Figure S4.

**Table 3:** Cancer-Causative Genes among the Oct4-Interacting Proteins

Gene	Mutation	Tissue	Cancer Type
CREB1	translocation	mesenchymal	clear cell sarcoma, angiomatoid fibrous histiocytoma
MITF	amplification	epithelial	melanoma
MSH6	Missense, nonsense, frameshift, splice site	epithelial	colorectal (somatic) colorectal, endometrial, ovarian (germline) non-polyposis colorectal cancer (hereditary)
POU5F1	translocation	mesenchymal	sarcoma
SMARCA4	frameshift, nonsense, missense	epithelial	NSCLC (non-small cell lung carcinoma)
TFE3	translocation	epithelial	papillary renal, alveolar soft part sarcoma, renal
TFEB	translocation	epithelial mesenchymal	renal, childhood epithelioid
TRIM24	translocation	blood	APL (acute promyelocytic leukemia)
TRIM33	translocation	epithelial	papillary thyroid

See also Figure S4.

In light of the central role of Oct4 in pluripotency and the cancer stem cell hypothesis, we investigated which of Oct4's physical interactors are misexpressed in cancer using the Oncomine human cancer expression database. A large fraction (60%) of the Oct4 interactors show misexpression in at least one cancer type, providing a degree of additional support to the connection between stem cell identity and cancer.

## Discussion

The characterization of protein-protein interactions is a very efficient strategy for understanding protein function and regulation. The development of high-affinity tags, including the TAP (Rigaut et al., 1999) and in vivo biotinylation tag (de Boer et al., 2003), in combination with advances in mass spectrometry that now allow protein identification with high sensitivity and accuracy, has recently produced several protein interaction network reports. However, most studies in the literature rely on cDNA overexpression driven by exogenous promoters or transgenic random integration approaches.

We report here an epitope-tagging strategy for the purification of protein complexes in mouse ESCs. We introduced the tag by recombineering into a full-length *Oct4*-containing BAC and then integrated this in a precise location in the mouse genome. This approach has the advantage of maintaining the endogenous promoter and therefore natural transcriptional regulation. The technology is amenable to high-throughput delivery, as recently demonstrated by random integration of tagged BAC transgenes (Poser et al., 2008), and should greatly facilitate systematic tagging of genes and analysis of protein complexes with roles in development in different contexts, be it in stem cells, differentiated cell types, or even mouse tissue (Fernández et al., 2009).

The affinity purification method described here is rapid, with the goal of capturing weak or short-lived interactions. Previous proteomic studies of Oct4 protein complexes have relied on lengthy single or tandem purifications from nuclear extracts with streptavidin capture (Wang et al., 2006) or anti-Oct4 antibodies (Liang et al., 2008) and yielded small data sets, very similar to our tandem purification data set. We identified all of the partners reported by the Liang study except Hdac2, and only five Oct4 partners found in the Wang study were not detected in our data set, maybe because of our use of whole extracts. Indeed, our approach has produced by far the most extensive analysis of Oct4-associated proteins to date.

**Figure 5:**
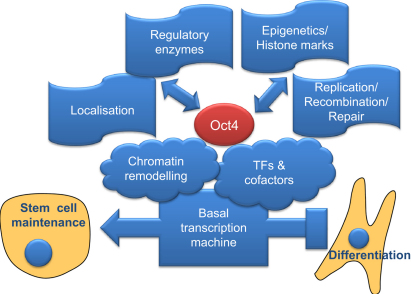
Schematic Model of the Oct4 Interactome

By using whole extracts, thereby not restricting the analysis to the nuclear environment, our data set encompasses diverse aspects of the life of Oct4, both nuclear and nonnuclear. The broad data set puts Oct4 at the center of diverse cellular processes that can have an impact on aspects of stem cell biology (Figure 5), the most interesting of which are discussed below.

Oct4 can both activate and repress transcriptional targets in mouse and human ESCs (Babaie et al., 2007, Loh et al., 2006). To date, Oct4 has been shown to be associated mainly with members of repressor complexes NuRD and SWI/SNF (Liang et al., 2008, Wang et al., 2006). We found both among our data set of Oct4-copurifying proteins. NuRD, a histone deacetylase complex, was the most prominent, further confirming this link. Sall4, a well-known Oct4 partner, and other members of the Spalt-like family of transcriptional cofactors have been shown to associate to NuRD (Lauberth and Rauchman, 2006), raising the possibility that they may bridge the interaction between Oct4 and NuRD. This hypothesis is also supported by the similar amounts in which they are detected in our experiments. We also found several subunits of the SWI/SNF nucleosome-remodeler complex, some of which have previously been linked to Nanog (Liang et al., 2008), confirming the link to this chromatin remodeling complex.

Also among Oct4 binding proteins we found various molecules involved in positive regulation of transcription, including several activators and coactivators and chromatin-modifying enzymes such as Myst2, a histone H4 acetyltransferase (Doyon et al., 2006, Sterner and Berger, 2000). In addition, we detected Ttf2, a component of the general transcription machinery, providing evidence of a physical link between pluripotency factors and basal transcription players. The Oct4 interactome included other basal DNA-process-related factors such as proteins involved in DNA replication, recombination, and repair. This could explain why many of the Oct4-interacting proteins are ubiquitously expressed in both differentiated and undifferentiated cells. Our experiments suggest that the interaction is not DNA mediated, given that copurification of DNA-binding proteins still occurs upon DNA elimination by benzonase.

Importantly, we have uncovered enzymes with a potential role in Oct4 regulation. Ogt is responsible for posttranslational addition of O-linked N-acetylglucosamine (O-GlcNAc), a regulatory protein modification similar to phosphorylation possibly working in concert with it (Kamemura and Hart, 2003). Oct4 is modified by O-GlcNAc in human ESCs (Webster et al., 2009), and Sp1, one of Oct4 partners, is too (Jackson and Tjian, 1988). A thorough analysis of O-GlcNAc modification in the Oct4 interactome might yield important insight into dynamic modulation of stem cell factors. Posttranslational modification of transcription factors and cofactors is proving to be a critical component of the regulation of gene transcription in general, and important specifically in stem cell biology (Brill et al., 2009, Van Hoof et al., 2009).

Half of Oct4-associated proteins seem to be directly regulated by transcription factors with key roles in stem cell pluripotency and/or reprogramming. This is also a characteristic of pluripotency networks derived from smaller data sets from different entry points (Orkin et al., 2008, Wang et al., 2006). This indicates that even in the expanded and functionally diverse network, this attribute still holds true, supporting a previously unsuspected role in stem cell biology for some of the proteins we identify here.

Expression of Oct4 decreases in a switch-like fashion as ESCs differentiate into lineage-specific cell types, including progenitor cells. Our analysis has uncovered 33 physical interactors of Oct4 that share this trend. Among these are several transcription factors, such as the DNA methyltransferase 3-like regulatory protein Dnmt3l, which stimulates genomic imprinting in germ cells (Bourc'his et al., 2001, Gowher et al., 2005). This is consistent with a recent report demonstrating that treatment with DNA methyltransferase inhibitors can improve the efficiency of the reprogramming process of differentiated cells (Mikkelsen et al., 2008). Therefore, the 33 interactors upregulated in ESCs and the transcription factors that regulate them might be interesting candidates whose expression could be manipulated to facilitate reprogramming.

We find that loss of function of most Oct4-associated genes studied to date results in embryonic or perinatal lethality, suggesting that many serve crucial functions in development. Interestingly, most Oct4-binding proteins linked to a human hereditary disorder (13 of 14), mostly developmental or cancer predisposition, give rise to a related phenotype when absent in the mouse. We find cancer-associated genes, either causal or predisposing, to be transcriptional regulators involved in processes relating to the cell cycle, differentiation, and DNA repair, acting through chromatin remodeling, signaling, or transcription factor activity. These results implicate the orthologs of Oct4-interacting proteins in roles in human development and cancer, and therefore the data presented here should be useful in elucidating their part in human disease.

In summation, the extensive systems-level analyses described here compiling data sets of currently available genome-wide studies provide an integrated vision of the Oct4 interactome. Detailed investigation of this information should facilitate the choice of candidate factors to test for roles in ESC maintenance, differentiation, and reprogramming and provide great insight into the transcriptional regulation of ESC biology.

## Experimental Procedures

### Generation of FTAP-Tagged *Oct4* ESCs

Full details are provided in Supplemental Experimental Procedures. In brief, the FTAP epitope tag (3×FLAG-2×TEV-CBP) sequence was synthesized as two DNA fragments by annealing overlapping complementary oligonucleotide molecules with PCR. The two fragments were cloned into a modified version of recombineering vector PL450 (Liu et al., 2003) for pCTR9 creation. The correctness of the FTAP tag within pCTR9 was confirmed by sequencing. Homology arms for recombineering were PCR amplified from the *Oct4* containing C57Black/6J derived BAC clone (RPCI 23-213M12) and cloned into the recombineering vector to create pCTS1 (Figure S1). The 5′ homology arm creates an in-frame fusion between the *Oct4* C-terminal coding sequence and the FTAP tag coding sequence, while deleting the stop codon. A fragment for recombineering the FTAP tag sequence into the *Oct4*-containing BAC (RPCI 23-213M12) was generated by digesting clone pCTS1. Correct recombination into *E. coli* DH10B containing BAC clone RPCI 23-213M12 was confirmed by Southern analysis of BAC DNA with homology arm-specific DNA probes for all six tagged BAC clones tested.

ESC cultures, electroporation, and mini-Southern-blot analysis of ESC clones were as described previously (Ramírez-Solis et al., 1993). Integration of single-copy BAC transgenes at the *Hprt* locus by recombinase-mediated cassette exchange (RMCE) has been described previously (Prosser et al., 2008). For RMCE integration of tagged *Oct4* BAC insert into *hprt^tm(rmce1)Brd^* allele of CCI18#1.6G, cells were cotransfected with pCAGGS-*Cre* (Araki et al., 1997) and the RPCI 23-213M12 BAC clone carrying an integrated copy of the FTAP tag cassette and neomycin resistance gene. Double-resistant colonies were isolated after successive selection with G418 (200 mg/ml) and 6-TG (10 μM). Site-specific BAC integration was very efficient, as verified by Southern analysis with *Hprt* flanking probes, with 19 of 23 double-resistant colonies showing correct single-copy integration. For removal of the selection cassette, the verified ESC clones were transfected with pCAGGS-*Flpe* (Schaft et al., 2001) and then selected with FIAU (200 nM). FIAU-resistant subclones were assessed for selection cassette deletion by Southern blotting. Absence of a hybridizing 5 kb fragment indicated successful deletion of the selection cassette. Transgenic clones were analyzed for expression of tagged *Pou5f1* by Western blotting, demonstrating that 60% of clones expressed the Oct4-FTAP fusion protein.

### Affinity Purification

Murine ESCs expressing Oct4-FTAP or wild-type control cells (AB2.2) were separated from feeders by trypsinization and incubation on gelatin-coated plates for 60 min. Whole-cell extracts were incubated with anti-FLAG M2 Dynal beads in buffer containing 150 mM NaCl and 0.1% NP-40 for 90 min at 4°C. Anti-FLAG Dynal beads were prepared by crosslinking M2 FLAG antibody (Sigma) to Protein G-Dynal beads (Invitrogen) in accordance with the manufacturer's instructions. Bound complexes were eluted with AcTEV protease (Invitrogen). For tandem affinity purification, the TEV eluate was incubated with calmodulin resin (Stratagene) for 60 min at 4°C. Elution was carried out by Ca chelation with EGTA. TEV or EGTA eluates were concentrated in Vivaspin 500 PES centrifugal filters (Vivascience), reduced with 1 mM DTT, and alkylated with 2 mM iodoacetamide prior to sample fractionation by polyacrylamide gel electrophoresis with Novex NuPAGE Bis-Tris 4%–12% gels (Invitrogen). Gels were stained with colloidal Coomassie (Sigma) according to Rowley (Rowley et al., 2000). Whole lanes were cut in 24 slices, destained completely, and digested with trypsin (sequencing grade, Roche). Peptides were extracted with 0.5% formic acid 50% acetonitrile and dried in a Speed Vac (Thermo).

### Immunoprecipitation and Western Blotting

Oct4 complexes were immunoprecipitated with an Oct4 antibody (Santa Cruz) coupled to Dynal-Protein G beads (Invitrogen). Immunoprecipitates were eluted by boiling in 1× LDS loading buffer (Invitrogen) and separated by LDS-PAGE (Invitrogen). Western blotting was carried out with antibodies from Abcam (Parp1, Sall4, and Myst2), Bethyl Laboratories (Chd4), or Santa Cruz (Oct4 and Hdac1).

### Mass Spectrometry and Data Analysis

Peptides were redissolved in 0.5% formic acid and analyzed with online nanoLC-MS/MS on a LTQ FT mass spectrometer (Thermo Fisher Scientific) coupled with an Ultimate 3000 Nano/Capillary LC System (Dionex). Samples were first loaded and desalted on a trap (0.3 mm id × 5 mm) at 25 μL/min with 0.1% formic acid for 5 min and then separated on an analytical column (75 μm id × 15 cm) (both PepMap C18, LC Packings) over a 30 min linear gradient of 4%–32% CH_3_CN/0.1% formic acid at 300 nL/min. The LTQ FT was operated in standard data-dependent acquisition. The survey scans (m/z 400–2000) were acquired on the FT-ICR at a resolution of 100,000 at m/z 400, and one microscan was acquired per spectrum. The three most abundant multiply charged ions with a minimal intensity of 1000 counts were subject to MS/MS in the linear ion trap at an isolation width of 3 Th. Dynamic exclusion width was set at ± 10 ppm for 45 s. The automatic gain control target value was regulated at 5E5 for FT and 1E4 for the ion trap, with maximum injection time at 1000 ms for FT and 200 ms for the ion trap, respectively.

The raw files were processed with BioWorks (Thermo). Database searches were performed with Mascot v.2.1 (Matrix Science) against the mouse IPI database (v. January 2009). The search parameters were: Trypsin/P with two missed cleavages, 10 ppm mass tolerance for MS, 0.5 Da tolerance for MS/MS, fixed modification Carbamidomethyl (C), and variable modifications of Acetyl (Protein N-term), Deamidated (NQ), Dioxidation (M), Formyl (N-term), Gln- > pyro-Glu (N-term Q), Methyl (E), and Oxidation (M). Decoy database searches were performed at the same time as the real searches, resulting in false discovery rates under 5%.

Only peptides with scores above 20 were used in the analysis. Protein identification required at least one high-confidence peptide (peptide score above identity threshold, e ≤ 0.05, length > 8 aas, precursor ion mass accuracy < 5 ppm where e ≥ 0.005, peptide hit rank 1, and delta peptide score > 10). There is increased risk of false discovery when a protein is identified by only one peptide. Thus, all peptides identifying a protein without additional support met the strict confidence requirements above and were manually verified. Precursor ion mass accuracies of these peptides are shown in Table S1. Mascot results were clustered to 95% protein homology to collapse highly homologous sequences corresponding to the same gene, and all lists for target and control purifications were compared in parallel. External contaminants (keratins, albumin, casein, and TEV protease) were excluded from the list. In the final list of Oct4-associated proteins we report only proteins identified in all three replicates. We have chosen one representative of each protein cluster, the one with the highest number of peptide matches, meaningful gene symbol, and highest molecular weight.

### Computational and Systems-Level Analysis

Orthologous human proteins were identified with the g:Profiler orthology search tool (Reimand et al., 2007) or NCBI BLASTP and aligned with the Needleman-Wunsch algorithm. For assessment of the degree of conservation between the Oct4-associated proteins and their orthologs, sequence identities of all mouse-to-human ortholog pairs of comparable sequence length in ENSEMBL release 57 were compared via a Mann-Whitney U test.

Domains were identified with Pfam 24.0, and genome-wide frequencies were calculated from domain annotations in UniProtKB/Swiss-Prot release 15.15 (Uniprot Consortium, 2010).

Mammalian Phenotype Ontology annotations were obtained from the Mouse Genome Informatics project (Blake et al., 2009), human disease associations were obtained from OMIM (Hamosh et al., 2005), and known cancer-causing mutations in genes were obtained from the Cancer Gene Census (Futreal et al., 2004). Student's t test was used for assessing the significance of the observed numbers of Oct4-associated genes with lethal phenotypes, disease, and cancer associations against 1000 random sets of 92 genes, in each case.

ChIP-on-chip data were obtained for Oct4 and eight other transcription factors (Kim et al., 2008). The significance of the number of Oct4-associated proteins regulated by these factors was assessed against 1000 random sets of 92 genes. The protein interaction network was generated with Cytoscape 2.6.3 (Cline et al., 2007), with a spring-embedded layout.

For the analysis of expression at different stages of differentiation, data were obtained for 43 mouse samples in StemBase (Sandie et al., 2009), originating from 16 studies with Affymetrix MOE430A microarray chips, as used in an Oct4 expression profiling study (Campbell et al., 2007) covering murine ESCs, embryonal carcinoma cell lines, and several early differentiated lineages. Expression data was available for 70 of the 92 Oct4-associated proteins. Where multiple probes were available, expression was averaged. Student's t test was used for identifying genes differentially expressed in ESCs as compared to more differentiated cell types (Bonferroni-corrected for multiple testing). Expression values were log2-transformed and color-coded as a gradient from blue (more than twice the standard deviation below the global microarray mean) via black (microarray mean) to yellow (more than twice the standard deviation above the mean). Average-linkage hierarchical clustering was performed to arrive at the final layout.

Data on significantly misexpressed genes was curated from the Oncomine human cancer expression database (Rhodes et al., 2007). Genes were considered mis-expressed below a p-value threshold of 10^−10^ (Bonferroni-corrected for multiple testing).

## Supplemental Information

Document S1. Four Figures, Table S2, and Supplemental Experimental Procedures

Table S1. Precursor Ion Mass Accuracy Mass accuracy of precursor ions of all peptides solely identifying a protein without additional support (“one-hit-wonders”). This table is related to Table 1.

Table S3. Domain Architectures of Oct4-Interacting Proteins PFAM domains are listed in the order they appear in each protein. This table is related to Table 1.

Table S4. Domain Occurrence, Function and Overrepresentation The number of occurrences of a certain domain within the Oct4-interacting set is given, as well as the number of distinct proteins bearing the domain. Domain functions were obtained from PFAM annotations. Fold enrichment was calculated by comparison of the domain composition of Oct4 partners to both the nuclear subset and complete set of SwissProt proteins with subcellular localization information. This estimates domain overrepresentation in the Oct4-associated group. Only domains significantly enriched are shown. This table is related to Table 1.

Table S5. Phenotypic Analysis List of phenotypes caused by spontaneous, induced, and/or genetically-engineered mutations in the genes encoding the Oct4-associated proteins identified in this study retrieved from the MGI database resource. This table is related to Figure 4.

Table S6. Master Table of Oct4-Interacting Proteins Summarizing All the Data from the Systems Analyses MW, molecular weight. Exp I, II, and IIIPep# show the number of unique peptides with scores above homology threshold in two independent experiments. AV_PROTSCORE is the protein score average between experiments I, II, and III. MGI phenotypes are labeled as follows: EL, embryonic lethality; PL, perinatal lethality; O, other; and NP, no phenotype detected. A blank cell means not studied. This table is related to Table 1.
