# Supplementary material for: An Expanded Oct4 Interaction Network: Implications for Stem Cell Biology, Development, and Disease
Source: Cell Stem Cell. 2010 Apr 2;6(4):382–95. doi: 10.1016/j.stem.2010.03.004 (PMC2860244; doi:10.1016/j.stem.2010.03.004)
Supplement: Document S1. Four Figures, Table S2, and Supplemental Experimental Procedures [file mmc1.pdf]

Supplemental Information

An Expanded Oct4 Interaction Network: Implications for Stem Cell Biology, Development and Disease

Mercedes Pardo, Benjamin Lang, Lu Yu, Haydn Prosser, Allan Bradley, M. Madan Babu, and Jyoti Choudhary

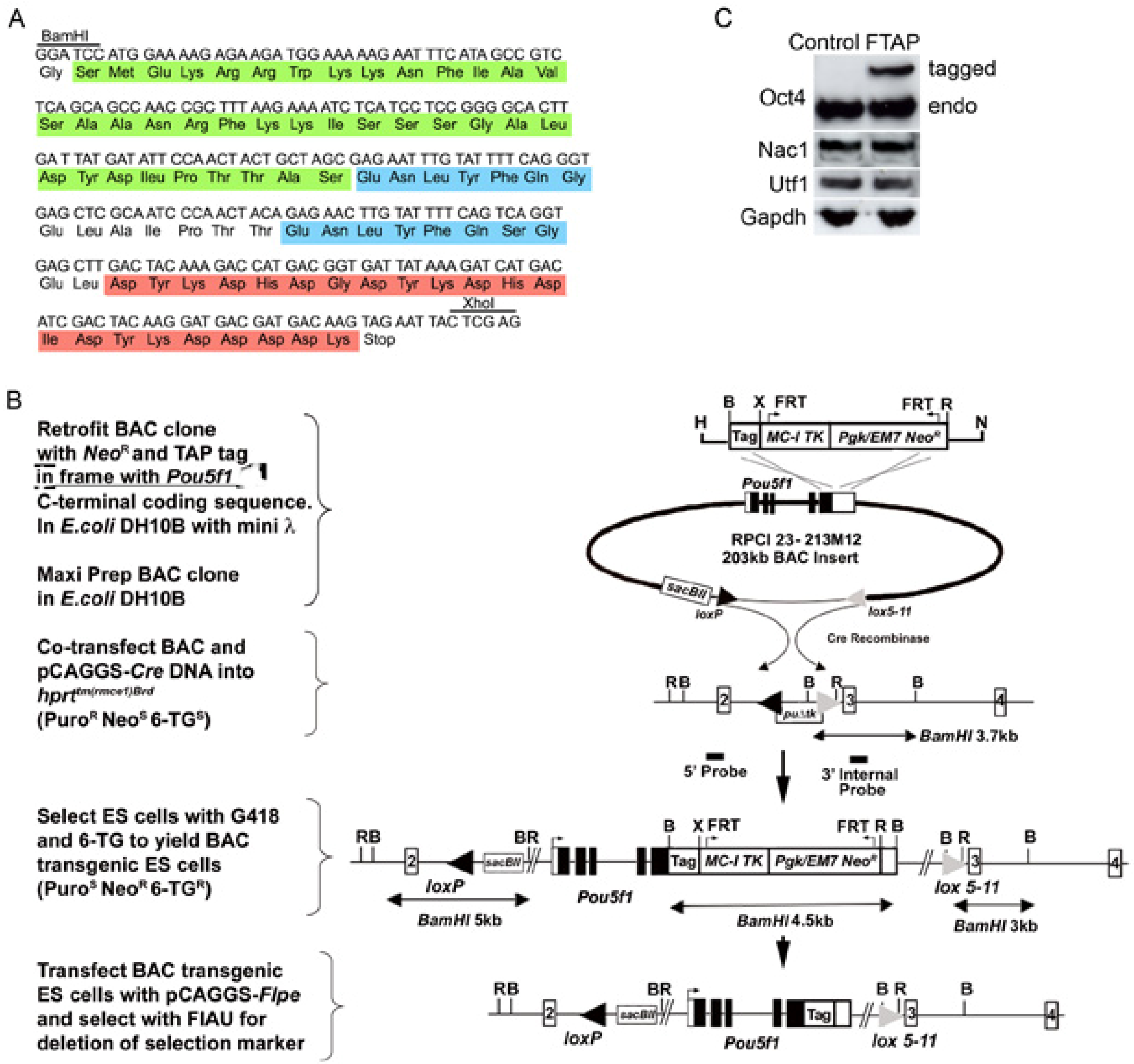

Figure S1, related to Figure 1. *Pou5f1* tagging strategy. A, DNA and amino acid sequence of the FTAP tag fragment within the recombineering vector pCTR9. The coding sequences of the constituent domains and epitopes (CBP (calmodulin binding peptide) in green, 2xTEV in blue, 3xFLAG in red), and the positions of the *BamHI* and *XhoI* cloning sites are indicated. **B,** Modification of the C-terminal coding

sequence of *Pou5f1* within BAC RPCI23-213M12 by recombineering of a FTAP tag and subsequent integration of the modified BAC insert into the *hprt* $^{tm(rmce1)Brd}$ locus are schematically depicted. X: *XhoI,* N: *NdeI*, R: *EcoRI*, B: *BamHI*, *Neo*/*Kan*^{R}: neomycin and kanamycin resistance gene, TK; *thymidine kinase* gene. The sensitivity (S-superscript) and resistance (R-superscript) of ES cell clones to the drugs puromycin (Puro), neomycin (Neo) and 6-thioguanine (6-TG) are indicated in the flow-diagram on the left. **C,** Western blots showing Oct4 expression (tagged and endogenous), and expression of markers of ES cells (Utf1, Nac1 and endogenous Oct4 itself). Gapdh was used as a loading control. The average ratio of endogenous Oct4 signal to tagged Oct4 (expressed from both alleles) is 3.032 (n=4, S.D.=0.39), as determined by pixel intensity analysis (ImageJ, NIH).

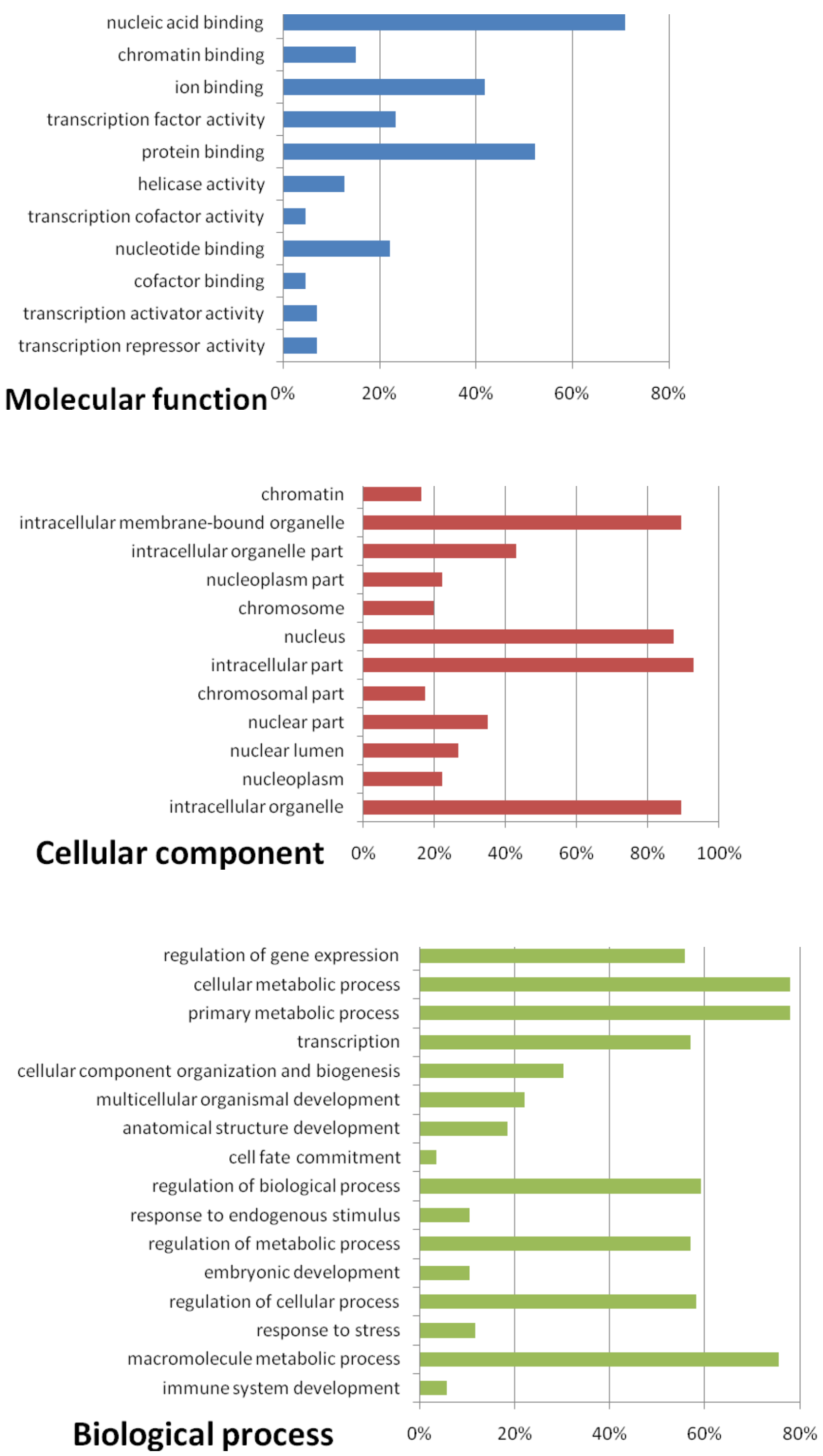

Figure S2, related to Table 1. Analysis of GO term enrichment of Oct4-associated proteins. In the GO Cellular Compartment category only terms with Bonferroni-corrected p < E-03 are shown for simplicity. Full data is shown in Table S2.

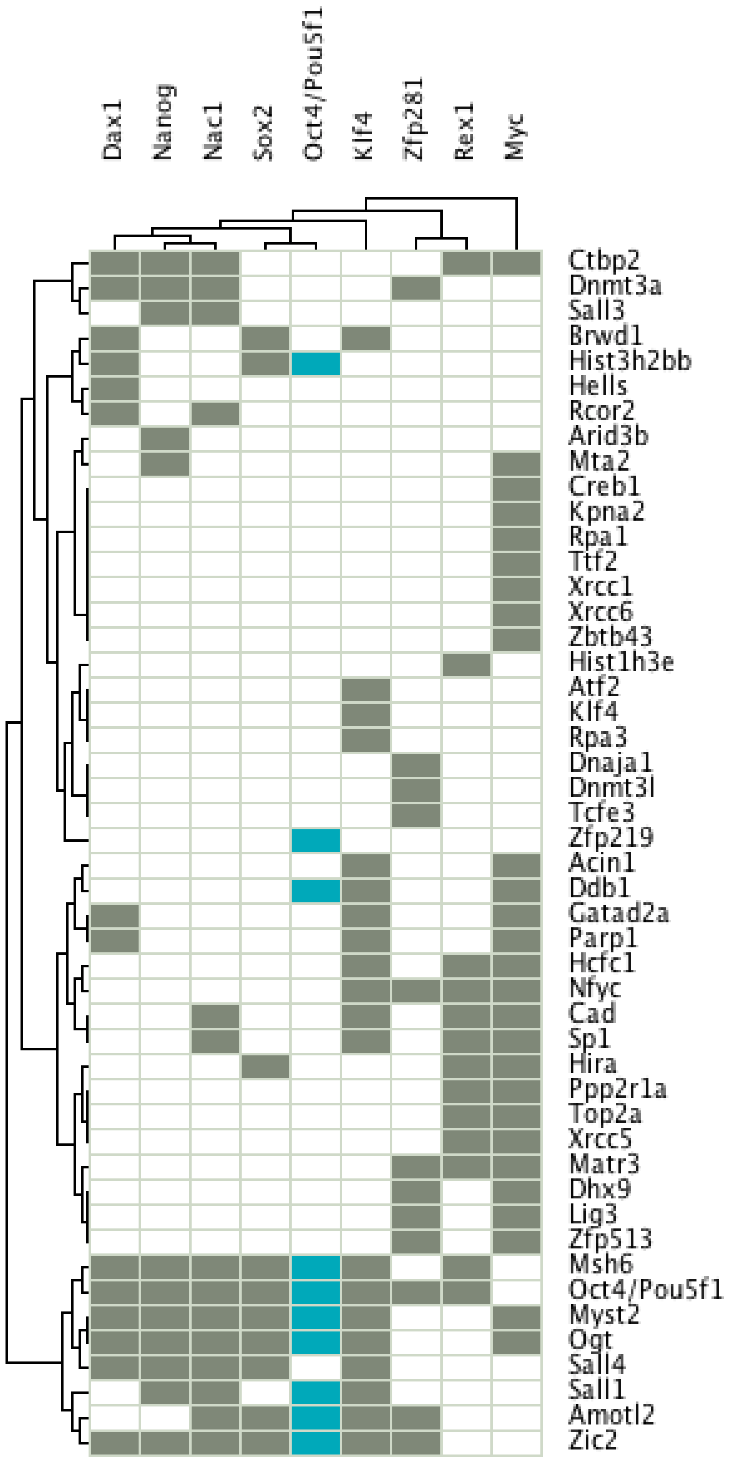

Figure S3, related to Figure 3. Regulation of the Oct4 "interactome". Matrix representation of promoter binding on Oct4-interacting genes (rows) by key stem cell transcription factors (columns).

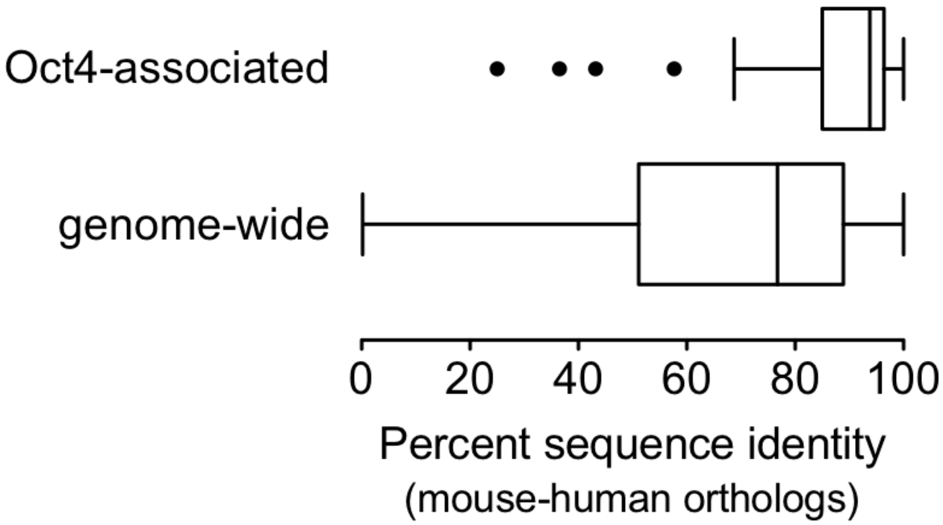

Figure S4, related to Table 2. Sequence identity of mouse-human ortholog pairs. Box plot with Tukey whiskers, showing sequence identity percentages between the identified Oct4-associated proteins and their human orthologs, as determined by global sequence alignment. The difference is significant at $p < 0.0001$. Genome-wide sequence identities for all mouse-to-human ortholog pairs contained in ENSEMBL are shown for comparison.

Supplemental Tables

Table S1, related to Table 1. Precursor ion mass accuracy. Mass accuracy of precursor ions of all peptides solely identifying a protein without additional support ("one-hit-wonders").

Table S2, related to Table 1. Functional category analysis for enriched GO and PANTHER terms among Oct4-associated proteins. A, Gene Ontology analysis using DAVID. The statistical analysis was carried out using the mouse genome as the reference dataset. Gene Ontology categories include Biological Process (BP), Cellular Component (CC) and Molecular Function (MF). **B**, PANTHER analysis for Pathway, Biological Process and Molecular Function enrichment, carried out in an analogous fashion.

A

Category	Identifier	Term	Count	%	Fold Enrichment	Bonferroni
MF_2	GO:0016564	transcription repressor activity	6	7.0%	6.79	0.179607
MF_2	GO:0016563	transcription activator activity	6	7.0%	5.46	0.397778
MF_2	GO:0048037	cofactor binding	4	4.7%	4.08	0.999746
MF_2	GO:0000166	nucleotide binding	19	22.1%	1.78	0.802838
MF_2	GO:0003712	transcription cofactor activity	4	4.7%	4.93	0.994351
MF_2	GO:0004386	helicase activity	11	12.8%	14.87	3.09E-07
MF_2	GO:0005515	protein binding	45	52.3%	1.51	0.072133
MF_2	GO:0003700	transcription factor activity	20	23.3%	4.20	1.49E-05
MF_2	GO:0043167	ion binding	36	41.9%	1.79	0.017447
MF_2	GO:0003682	chromatin binding	13	15.1%	21.63	6.82E-11
MF_2	GO:0003676	nucleic acid binding	61	70.9%	3.56	1.14E-22
CC_4	GO:0043229	intracellular organelle	77	89.5%	1.77	1.15E-13
CC_4	GO:0005958	DNA-dependent protein kinase complex	2	2.3%	172.91	0.997384
CC_4	GO:0000118	histone deacetylase complex	5	5.8%	30.88	0.009403
CC_4	GO:0005654	nucleoplasm	19	22.1%	5.32	4.48E-06
CC_4	GO:0031981	nuclear lumen	23	26.7%	5.25	7.17E-08
CC_4	GO:0044428	nuclear part	30	34.9%	4.63	5.55E-10
CC_4	GO:0044427	chromosomal part	15	17.4%	8.62	7.26E-07
CC_4	GO:0044424	intracellular part	80	93.0%	1.55	5.47E-12
CC_4	GO:0005634	nucleus	75	87.2%	3.08	2.67E-29
CC_4	GO:0031965	nuclear membrane	3	3.5%	7.52	1
CC_4	GO:0000228	nuclear chromosome	5	5.8%	9.20	0.653248
CC_4	GO:0005694	chromosome	17	19.8%	8.21	7.52E-08
CC_4	GO:0044454	nuclear chromosome part	5	5.8%	10.94	0.426721
CC_4	GO:0044453	nuclear membrane part	3	3.5%	7.86	1
CC_4	GO:0016514	SWI/SNF complex	2	2.3%	57.64	1
CC_4	GO:0044451	nucleoplasm part	19	22.1%	5.66	1.66E-06
CC_4	GO:0043232	intracellular non-membrane-bound organelle	20	23.3%	2.14	0.549728
CC_4	GO:0044446	intracellular organelle part	37	43.0%	2.45	2.86E-05
CC_4	GO:0043231	intracellular membrane-bound organelle	77	89.5%	2.00	1.27E-17
CC_4	GO:0000785	chromatin	14	16.3%	13.83	9.13E-09
BP_2	GO:0002520	immune system development	5	5.8%	3.45	0.999898

BP_2	GO:0043170	macromolecule metabolic process	65	75.6%	2.01	3.56E-12
BP_2	GO:0006950	response to stress	10	11.6%	2.32	0.985469
BP_2	GO:0050794	regulation of cellular process	50	58.1%	2.55	1.14E-10
BP_2	GO:0009790	embryonic development	9	10.5%	3.83	0.296769
BP_2	GO:0019222	regulation of metabolic process	49	57.0%	3.57	2.27E-16
BP_2	GO:0009719	response to endogenous stimulus	9	10.5%	5.91	0.019995
BP_2	GO:0050789	regulation of biological process	51	59.3%	2.36	1.10E-09
BP_2	GO:0045165	cell fate commitment	3	3.5%	5.66	1
BP_2	GO:0048856	anatomical structure development	16	18.6%	1.51	1
BP_2	GO:0007275	multicellular organismal development	19	22.1%	1.63	0.995545
BP_2	GO:0016043	cellular component organization and biogenesis	26	30.2%	1.88	0.174739
BP_2	GO:0006350	transcription	49	57.0%	3.97	2.26E-18
BP_2	GO:0044238	primary metabolic process	67	77.9%	1.81	1.33E-10
BP_2	GO:0044237	cellular metabolic process	67	77.9%	1.81	1.34E-10
BP_2	GO:0010468	regulation of gene expression	48	55.8%	3.77	7.93E-17

B

Category	Term	% of Genes	Count	Expected	p-Value
PW	Wnt signaling pathway	8.60%	8	1.3	7.2E-03
BP	Nucleoside, nucleotide and nucleic acid metabolism	73.12%	68	12.0	9.0E-39
BP	mRNA transcription	48.39%	45	7.1	1.2E-23
BP	mRNA transcription regulation	36.56%	34	5.2	7.0E-17
BP	DNA metabolism	13.98%	13	1.0	5.3E-09
BP	Chromatin packaging and remodeling	11.83%	11	0.8	1.2E-07
BP	DNA repair	8.60%	8	0.5	6.1E-06
BP	DNA recombination	4.30%	4	0.1	2.4E-03
BP	Other mRNA transcription	3.23%	3	0.1	6.3E-03
MF	Nucleic acid binding	64.52%	60	11.3	9.0E-31
MF	Transcription factor	33.33%	31	7.3	5.3E-11
MF	Zinc finger transcription factor	17.20%	16	3.3	2.8E-05
MF	Helicase	12.90%	12	0.5	6.5E-11
MF	DNA helicase	11.83%	11	0.2	3.3E-13
MF	Chromatin/chromatin-binding protein	11.83%	11	0.5	8.7E-10
MF	Histone	5.38%	5	0.4	5.5E-03
MF	DNA methyltransferase	3.23%	3	0.1	3.7E-03

Table S3, related to Table 1. Domain architectures of Oct4-interacting proteins. PFAM domains are listed in the order they appear in each protein.

(Attached as separate Excel file)

Table S4, related to Table 1. Domain occurrence, function and over-representation. The number of occurrences of a certain domain within the Oct4 interacting set is given, as well as the number of distinct proteins bearing the domain. Domain functions were obtained from PFAM annotations. Fold enrichment was calculated comparing the domain composition of Oct4 partners to both the nuclear subset and complete set of SwissProt proteins with subcellular localisation information. This estimates domain overrepresentation in the Oct4-associated group. Only domains significantly enriched are shown.

(Attached as separate Excel file)

Table S5, related to Figure 4. Phenotypic analysis. List of phenotypes caused by spontaneous, induced, and/or genetically-engineered mutations in the genes encoding the Oct4-associated proteins identified in this study retrieved from the MGI database resource.

(Attached as separate Excel file)

Table S6, related to Table 1. Master table of Oct4 interacting proteins summarising all the data from the systems analyses, related to Table 1. MW, molecular weight. Exp I, II and IIIPep# show number of unique peptides with scores above homology threshold in three independent experiments. AV_PROTSCORE is the protein score average between experiments I, II and III. MGI phenotypes are labelled as follows: EL Embryonic lethality, PL Perinatal lethality, O Other, NP No phenotype detected, blank means not studied.

(Attached as separate Excel file)

Supplemental Experimental Procedures

Generation of c-terminal FTAP tag recombineering constructs

The recombineering vector PL450 (Liu et al., 2003) was modified at the *BstBI* site by insertion of a *SrfI* restriction site as a double stranded oligonucleotide linker (pCOM). An *RsrII* fragment from the vector pKO SelectTK (Lexicon Genetics) was made blunt-ended by filling-in the overhang with Klenow and ligated into the modified PL450 at the *SrfI* site (pCOI1). The polylinker of the recombineering vector (pCOI1) was

modified by cloning of a double stranded oligo nucleotide linker (*BamHI* overhang/ *XhoI/ BamHI/ SacII*) to generate a unique *XhoI* site.

The FTAP epitope tag (3xFLAG-2xTEV-CBP) sequence was synthesized as two DNA fragments by annealing two overlapping complementary oligonucleotide molecules using PCR with HiFi Supermix (Invitrogen). Restriction sites for generating sticky ends by digestion for ligation are indicated by underlining.

5’ FTAP fragment oligos:

5’-

AAAAAAAAGGATCCATGGAAAAGAGAAGATGGAAAAAGAATTTCATAGCCGTCTCAGCAGCC

AACCGCTTTAAGAAAATCTCATCCTCCGGGGC-3’ and

5’-

AAAAAAAAAAAAAAAGAGCTCACCCTGAAAATACAAATTCTCGCTAGCAGTAGTTGGAATATC

ATAATCAAGTGCCCCGGAGGATGAGATTTTCTTAAAG-3’

3’ FTAP fragment oligos:

5’-

AAAAAAAAAAAAGAGCTCGCAATCCCAACTACAGAGAACTTGTATTTTCAGTCAGGTGAGCTT

GACTACAAAGACCATGACGGTGATT-3’ and

5’-

AAAAAAAAACTCGAGTAATTCTACTTGTCATCGTCATCCTTGTAGTCGATGTCATGATCTTTAT

AATCACCGTCATGGTCTTTGTAG-3’

The filled-in double-stranded products were double restriction digested with *BamHI/SacI* for the 5’ FTAP tag fragment and *SacI/XhoI* for the 3’ FTAP tag fragment. The two fragments were cloned into the *BamHI/XhoI* digested recombineering vector (pCOI1) as a three way ligation to create pCTR9 (Figure S1B). The correctness of the FTAP tag within pCTR9 was confirmed by sequencing.

Generation of *Pou5f1* recombineering construct

Homology arms for recombineering were PCR amplified from the *Oct4* containing C57Black/6J derived BAC clone (RPCI 23-213M12) using the following oligonucleotides primers (restriction sites for generating

sticky ends by digestion for ligation are indicated by underlining; NCBI m37 assembly chromosomal locations of the oligonucleotide sequence are indicated in brackets):

5’ arm: ACCCAAGCTTCCCCACCGCCGCCACCGCTGA (Chr. 17:35,647,160 to 35,647,180bp) and TGGTGGATCCGTTTGAATGCATGGGAGAGC (Chr. 17: 35,647,474 to 35,647,493bp).

3’ arm: ACCAGAATTCCCTGGGGATGCTGTGAGCCAA (Chr. 17:35,647,510 to 35,647,530bp) and GAATTCCCATAGTGGTTTCAATGGCGCCTGT (Chr.17: 35,647,807 to 35,647,823bp).

The PCR products for the 5’ and 3’ homology were double-restriction digested with *HindIII/BamHI* and *EcoRI/NdeI* respectively and were sequentially cloned into the corresponding restriction sites of the recombineering vector to create pCTS1 (Figure S1). The 5’ homology arm creates an in-frame fusion between the *Oct4* C-terminal coding sequence and the FTAP tag coding sequence, whilst deleting the stop codon.

Recombineering reaction

E. coli DH10B containing BAC clone RPCI 23-213M12 were made competent for recombineering by electroporation the miniλ prophage and selecting overnight at 32°C on LB-agar plates with tetracycline (12.5μg/ml) and chloramphenicol (20μg./ml) (Court et al., 2003). A fragment for recombineering the FTAP tag sequence into the Oct4-containing BAC (RPCI 23-213M12) was generated by digesting clone pCTS1 with *HindIII*/*NdeI*. Correct recombination was confirmed by Southern analysis of BAC DNA using homology arm-specific DNA probes for all 6 tagged BAC clones tested.

Integration of modified BAC clones into the *Hprt* locus of ES cells by recombinase-mediated cassette exchange (RMCE)

ES cell cultures, electroporation and mini-Southern-blot analysis of ES cell clones were as described (Ramirez-Solis et al., 1993). The process for integrating single copy BAC transgenes at the *Hprt* locus by RMCE has been described previously (Prosser et al., 2008). For RMCE integration of tagged *Oct4* BAC insert into $hprt^{tm(rmce1)Brd}$ allele of CCI18#1.6G, cells were co-transfected by electroporation (Biorad; 500 μF, 230 V) with pCAGGS-*Cre* (Araki et al., 1997) and the RPCI 23-213M12 BAC clone carrying an integrated copy of the FTAP tag cassette and neomycin resistance gene. Double resistant colonies were isolated after selection with G418 (200 μg/ml) for 5-6 d, and subsequent selection with 6-TG (10 μM) for 3-

4 d. Site specific BAC integration was very efficient, as verified by Southern analysis using *Hprt* flanking probes, with 19 of 23 double resistant colonies showing correct single copy integration. For removal of the selection cassette the verified ES cell clones were transfected with pCAGGS-*Flpe* (Schaft et al., 2001) followed by selection with FIAU (200nM) for 5 dFIAU resistant subclones from the Flpe treated plates were isolated and expanded for assessment of selection cassette deletion by Southern blot. Genomic DNA was digested with an *XhoI/BamHI* double digest and Southern blot hybridized with an MC1-tk probe. Absence of a hybridizing 5kb fragment was indicated successful deletion of the selection cassette.

Supplemental References

Araki, K., Imaizumi, T., Okuyama, K., Oike, Y., and Yamamura, K. (1997). Efficiency of recombination by Cre transient expression in embryonic stem cells: comparison of various promoters. Journal of biochemistry *122*, 977-982.

Court, D.L., Swaminathan, S., Yu, D., Wilson, H., Baker, T., Bubunenko, M., Sawitzke, J., and Sharan, S.K. (2003). Mini-lambda: a tractable system for chromosome and BAC engineering. Gene *315*, 63-69.

Liu, P., Jenkins, N.A., and Copeland, N.G. (2003). A highly efficient recombineering-based method for generating conditional knockout mutations. Genome Res *13*, 476-484.

Prosser, H.M., Rzadzinska, A.K., Steel, K.P., and Bradley, A. (2008). Mosaic complementation demonstrates a regulatory role for myosin VIIa in actin dynamics of stereocilia. Mol Cell Biol *28*, 1702-1712.

Ramirez-Solis, R., Zheng, H., Whiting, J., Krumlauf, R., and Bradley, A. (1993). Hoxb-4 (Hox-2.6) mutant mice show homeotic transformation of a cervical vertebra and defects in the closure of the sternal rudiments. Cell *73*, 279-294.

Schaft, J., Ashery-Padan, R., van der Hoeven, F., Gruss, P., and Stewart, A.F. (2001). Efficient FLP recombination in mouse ES cells and oocytes. Genesis *31*, 6-10.
